# Vitamin D Genetics Beyond Serum 25(OH)D: *VDR* rs2228570 (*FokI*) Polymorphism, Inflammation, and Quality of Life in Orthopedic Patients

**DOI:** 10.3390/nu17182926

**Published:** 2025-09-11

**Authors:** Dariusz Larysz, Remigiusz Recław, Aleksandra Suchanecka, Wojciech Dziurawiec, Rafał Tkacz, Aleksandra Strońska-Pluta, Krzysztof Chmielowiec, Anna Grzywacz, Jolanta Chmielowiec

**Affiliations:** 1 Department of Trauma and Orthopaedic Surgery, 109th Military Hospital with Polyclinic, Ministry of National Defense, ul. Ksiedza Piotra Skargi 9/11, 71-422 Szczecin, Poland; dariuszlarysz@hotmail.com (D.L.); wojciechdziurawiec@hotmail.com (W.D.); robert.tkacz@hotmail.com (R.T.); 2 Independent Laboratory of Genetics and Behavioral Epigenetics, Pomeranian Medical University in Szczecin, Powstańców Wielkopolskich 72 St., 70-111 Szczecin, Poland; remigiusz.reclaw@pum.edu.pl (R.R.); aleksandra.suchanecka@pum.edu.pl (A.S.); aleksandra.stronska@pum.edu.pl (A.S.-P.); 3 Department of Medical Sciences and Public Health, Gdansk University of Physical Education and Sport, Kazimierza Górskiego 1 St., 80-336 Gdansk, Poland; 4 Department of Hygiene and Epidemiology, Collegium Medicum, University of Zielona Góra, 28 Zyty St., 65-046 Zielona Gora, Poland; chmiele@vp.pl (K.C.); chmiele1@o2.pl (J.C.)

**Keywords:** vitamin D receptor (*VDR*), rs2228570, *FokI* polymorphism, orthopedic surgery, blood biochemical markers, quality of life

## Abstract

Background: Vitamin D receptor (VDR) polymorphisms may influence immune regulation and musculoskeletal health, but their perioperative role is not well understood. This study investigated the rs2228570 (*FokI*) variant in relation to inflammatory, hematologic, and patient-reported outcomes in orthopedic patients. Methods: We genotyped 300 orthopedic patients and 200 healthy controls using real-time PCR. Regression models in patients adjusted for age and body mass index (BMI) examined associations between rs2228570 genotypes and laboratory as well as clinical outcomes. Results: The CC genotype (homozygous cytosine) was associated with higher white blood cell count (β = 0.52, *p* = 0.0435), higher lymphocyte count (β = 0.26, *p* = 0.00025), higher hemoglobin (β = 0.57, *p* = 0.00197), and higher hematocrit (β = 1.42, *p* = 0.01102). The TT genotype (homozygous thymine) was associated with higher C-reactive protein (β = 10.90, *p* = 0.00329), lower mean corpuscular volume (β = −1.63, *p* = 0.04909), and higher health-related quality of life assessed by the 36-Item Short-Form Health Survey (SF-36) (β = 6.31, *p* = 0.00009). Conclusions: The rs2228570 polymorphism in the VDR gene is associated with distinct perioperative inflammatory, hematologic, and patient-reported profiles. These findings support the potential clinical utility of VDR genotyping, in combination with routine laboratory tests, to refine perioperative risk stratification and guide personalized rehabilitation in orthopedic patients.

## 1. Introduction

Vitamin D is a secosteroid hormone essential for calcium–phosphate balance, skeletal integrity, and immune regulation [1,2]. While its classical role in bone metabolism is well established, ongoing research has revealed broader biological actions [2,3]. At the same time, the clinical implications of vitamin D status remain a matter of debate, with inconsistencies between circulating 25-hydroxyvitamin D_3_ levels and functional or health outcomes frequently reported. This controversy has drawn attention to the role of the vitamin D receptor (*VDR*)—a nuclear transcription factor that mediates the actions of 1,25-dihydroxyvitamin D_3_—whose genetic variability may critically influence downstream physiological responses and help explain these discrepancies [1,2].

The *VDR* gene is located on chromosome 12q13.11 and spans approximately 100 kb of genomic DNA, with about 75 kb encompassing the coding exons [4,5,6]. Several single nucleotide polymorphisms (SNPs) within the *VDR* gene have been identified and investigated for their potential influence on receptor activity and downstream physiological effects [7]. Among the most studied *VDR* variants, the rs2228570 polymorphism, also known as *FokI* (C/T), is located in exon 2 near the translation initiation site of the *VDR* gene. This variant creates or abolishes an alternative upstream start codon, resulting in a longer or shorter isoform of the VDR protein [8,9]. Functional studies suggest that the shorter isoform may exhibit higher transcriptional activity, potentially modulating the expression of vitamin D-responsive genes and influencing susceptibility to various diseases, including osteoporosis, autoimmune disorders, certain cancers, and other health conditions [8,9,10,11,12].

The present study aimed to investigate the association between the rs2228570 *FokI* polymorphism of the *VDR* gene and selected biochemical blood parameters, as well as self-reported quality of life, in patients undergoing orthopedic surgery. Given the potential role of *VDR* genetic variation in musculoskeletal health, immune regulation, and recovery potential, we hypothesized that specific rs2228570 genotypes may be associated with distinct biochemical profiles and quality-of-life scores in this patient population. To address this, we analyzed genotype distribution, tested for Hardy–Weinberg equilibrium, and assessed the relationship between the polymorphism, biochemical markers, and SF-36 questionnaire results, controlling for demographic and anthropometric variables such as age and body mass index (BMI). The rs2228570 polymorphism has been associated with diverse health conditions (e.g., metabolic dysfunction, osteoporosis, sleep impairments) and may also influence inflammatory markers and musculoskeletal outcomes relevant in postoperative recovery [11,13,14,15]. However, the relationship between vitamin D status, *VDR* genetic variability, and clinical outcomes remains controversial, with inconsistencies across studies and outcomes. Understanding the genotype–phenotype correlations involving this polymorphism may therefore provide new insight into why serum 25(OH)D alone does not fully capture vitamin D’s biological and clinical impact and could help identify subgroups of patients who benefit from tailored interventions [16].

By integrating genetic, biochemical, and patient-reported outcomes, this study offers a multidimensional perspective on the potential role of the *VDR* rs2228570 polymorphism in orthopedic patients [17]. This approach may enhance our understanding of genotype–phenotype relationships [18] and contribute to the identification of biomarkers relevant for prognosis, rehabilitation, and personalized care [19]. The following sections describe the study design, participant characteristics, genotyping, outcome measures, and statistical methods.

In orthopedic perioperative care, low-grade inflammation and hematologic dynamics are central to recovery trajectories and complication risk. Because rs2228570 (*FokI*) alters the structure and activity of the vitamin D receptor, it may modulate immune responses and erythropoiesis in ways that are particularly relevant during the perioperative period. Despite accumulating evidence linking this variant to inflammatory and musculoskeletal outcomes, its role in surgical patients remains poorly understood. Therefore, we investigated associations between rs2228570 genotypes and perioperative inflammatory markers, hematologic parameters, and health-related quality of life in orthopedic patients. By integrating genotyping with routine laboratory measures, this study aims to address a critical gap and highlight the potential clinical utility of VDR polymorphism analysis for risk stratification and personalized recovery pathways.

## 2. Materials and Methods

### 2.1. Participants

A total of 500 individuals participated in the study. The study group comprised 300 patients undergoing orthopedic surgery (men: n = 113, 37.7%; women: n = 187, 62.3%), while the control group included 200 healthy individuals with no history of musculoskeletal disorders or prior orthopedic interventions (men: n = 61, 30.5%; women: n = 138, 69.5%). The mean age of the orthopedic patients was 66.7 years, whereas the mean age in the control group was 30.7 years. Although there was a substantial age difference between groups, age was included as a covariate in the multiple regression models to control for its potential confounding effect. The orthopedic patient group included individuals scheduled for elective procedures such as hip or knee arthroplasty, fracture fixation, or corrective osteotomy. Patients were recruited from the Orthopedics Department of the 109th Military Hospital with Polyclinic in Szczecin, Poland, and met the inclusion criteria of being over 18 years of age, having no active malignancy, infectious, or autoimmune disease, and providing written informed consent to participate. The control group consisted primarily of local community members, as well as university medical staff and students, with a similar sex distribution but differing in age due to the clinical nature of the study group.

### 2.2. Measures

Biochemical blood parameters were assessed in all participants and included: white blood cell count (WBC, ×10^9^/L), lymphocyte count (LYM, ×10^9^/L), neutrophil count (NEU, ×10^9^/L), monocyte count (MONO, ×10^9^/L), eosinophil count (EOS, ×10^9^/L), basophil count (BASO, ×10^9^/L), hemoglobin concentration (HGB, g/dL), hematocrit percentage (HCT, %), mean corpuscular volume (MCV, fL), mean corpuscular hemoglobin (MCH, pg), mean corpuscular hemoglobin concentration (MCHC, g/dL), platelet count (PLT, ×10^9^/L), platelet distribution width (PDW, %), mean platelet volume (MPV, fL), plateletcrit (PCT, %), C-reactive protein (CRP, mg/L), serum 25-hydroxyvitamin D_3_ concentration (25(OH)D_3_, ng/mL), glycated hemoglobin (HbA1c, %), and serum creatinine (µmol/L). In Table 4, full names of parameters are presented alongside their abbreviations and measurement units, whereas in Tables 5 and 6 only abbreviations are used for brevity.

Body mass index (BMI, kg/m^2^) was calculated as body weight in kilograms divided by the square of height in meters.

Health-related quality of life was assessed using the Short Form Health Survey (SF-36), a validated 36-item questionnaire covering eight domains: physical functioning, role limitations due to physical health, bodily pain, general health, vitality, social functioning, role limitations due to emotional problems, and mental health.

### 2.3. Genotyping

Genomic DNA was isolated from peripheral venous blood using standard procedures. The extraction was performed according to the manufacturer’s protocol (ROCHE, Basel, Switzerland) and in compliance with the company’s quality control standards. The selection of reagents, primers, and probes was based on the specifications provided in the ROCHE real-time PCR methodology for single nucleotide polymorphism (SNP) detection.

The rs2228570 *FokI* (C/T) polymorphism in the vitamin D receptor (*VDR*) gene was determined using the real-time PCR technique with allele-specific fluorescent probes. Following amplification, melting curve analysis was performed for each sample by plotting the fluorescence signal against the temperature. Distinct melting peaks were used to identify alleles: The C allele was detected at approximately 57.5 °C and the T allele at approximately 63.8 °C. All reactions included negative controls (no-template controls) and selected samples were regenotyped to verify accuracy.

### 2.4. Statistics

The concordance between genotype frequency distribution and Hardy–Weinberg equilibrium (HWE) was tested using the HWE software (https://wpcalc.com/en/equilibrium-hardy-weinberg/, accessed on 12 June 2025). The normality of variable distribution was assessed using the Kolmogorov–Smirnov test. Data for normally distributed variables are presented as the mean ± standard deviation (SD), while non-normally distributed variables are presented as the mean with the lower quartile [Q1] and upper quartile [Q3]. Differences in blood parameters between genders were assessed using Student’s *t*-test for normally distributed variables and the Mann–Whitney U test for non-normally distributed variables. For categorical variables, differences were evaluated using the chi-square test. The association between the rs2228570 *FokI* gene polymorphism and blood biochemical parameters, as well as SF-36 Quality of Life Questionnaire scores, was analyzed using multiple regression. The dependent variables included blood parameters and SF-36 scores in orthopedic patients, while the independent variables were age, body mass index (BMI), and the rs2228570 *FokI* gene polymorphism. For the rs2228570 *FokI* polymorphism, a dummy variable was created, with CT heterozygotes serving as the reference category and homozygotes representing the effect on the dependent variables. A *p*-value < 0.05 was considered statistically significant. All analyses were performed using STATISTICA 13 (Tibco Software Inc., Palo Alto, CA, USA) and PQStat (version 1.8.6, Poznań, Poland) for Windows (Microsoft Corporation, Redmond, WA, USA).

## 3. Results

The genotype frequency distribution in the orthopedic patient group was consistent with the expectations of Hardy–Weinberg equilibrium (HWE). A similar adherence to HWE was observed in the control group (Table 1).

There was no statistically significant difference in the distribution of rs2228570 *FokI* genotypes between the orthopedic surgery group and the control group (CC 0.32 vs. CC 0.30; CT 0.50 vs. CT 0.50; TT 0.17 vs. TT 0.19; χ^2^ = 0.3134, *p* = 0.8549). Similarly, no statistically significant difference was observed in the allele frequencies of the rs2228570 *FokI* polymorphism between the two groups (C 0.57 vs. C 0.56; T 0.43 vs. T 0.44; χ^2^ = 0.2995, *p* = 0.5842; Table 2).

No statistically significant differences were observed in the frequency of the rs2228570 *FokI* polymorphism among orthopedic surgery patients when stratified by sex (χ^2^ = 0.9645, *p* = 0.6174), smoking status (χ^2^ = 1.6151, *p* = 0.4460), diagnosed hypertension (χ^2^ = 2.7394, *p* = 0.2542), or diabetes mellitus (χ^2^ = 0.2426, *p* = 0.8857; Table 3).

The general characteristics of the orthopedic surgery patient group, including age, BMI, blood biochemical parameters, and the SF-36 quality of life questionnaire scores, are presented in Table 4.

**Table 4:** Descriptive statistics of biochemical parameters and SF-36 quality of life questionnaire scores in orthopedic surgery patients.

	M (SD)/[Q1:Q3] *	Minimum	Maximum
Age	66.70 (10.08)	27.00	87.00
Body mass index (BMI)	29.17 (4.97)	17.93	48.42
White blood cells (WBC, ×10^9^/L)	6.99 (1.96)	2.40	14.76
Lymphocytes (LYM, ×10^9^/L)	1.71 (0.56)	0.60	4.42
Neutrophils (NEU, ×10^9^/L)	4.58 (1.69)	1.13	12.44
Monocytes (MONO, ×10^9^/L)	0.52 (0.17)	0.05	1.23
Eosinophils (EOS, ×10^9^/L)	0.12 [0.05:0.17] *	0.00	0.73
Basophils (BASO, ×10^9^/L)	0.04 [0.02:0.05] *	0.00	0.10
Hemoglobin (HGB, g/dL)	13.82 (1.44)	9.10	17.90
Hematocrit (HCT, %)	41.24 (4.38)	14.70	52.80
Mean corpuscular volume (MCV, fL)	90.59 (5.16)	76.50	120.30
Mean corpuscular hemoglobin (MCH, pg)	30.19 (2.16)	20.80	41.00
Mean corpuscular hemoglobin concentration (MCHC, g/dL)	33.36 (0.98)	28.40	36.60
Platelet count (PLT, ×10^9^/L)	257.27 (73.33)	12.00	717.00
Platelet distribution width (PDW, %)	13.35 [10.90:13.50] *	8.70	36.9
Mean platelet volume (MPV, fL)	11.53 [9.80:11.10] *	8.60	14.7
Plateletcrit (PCT, %)	0.43 [0.22:0.31] *	0.09	00.68
C-reactive protein (CRP, mg/L)	7.72 [0.99:4.49] *	0.05	211.55
25-hydroxyvitamin D3 (ng/mL)	32.70 [20.40:40.70] *	0.35	122.00
Glycated hemoglobin (HbA1c, %)	5.88 [5.38:6.00] *	4.72	9.85
Creatinine (µmol/L)	79.78 [64.00:86.00] *	46.00	282.00
SF-36 quality of life score	96.05 (10.19)	54.00	122.00

M—arithmetic mean; SD—standard deviation; Q1—lower quartile; Q3—upper quartile; *—variables do not follow a normal distribution according to the Kolmogorov–Smirnov test.

Table 5 presents differences in biochemical parameters and SF-36 quality of life questionnaire scores among orthopedic surgery patients stratified by sex. Compared with women, men exhibited significantly higher body mass index (29.94 vs. 28.71; t = 2.0890, *p* = 0.03755), monocyte count (0.57 vs. 0.49; t = 3.8819, *p* = 0.00013), hemoglobin concentration (14.51 vs. 13.40; t = 6.9779, *p* < 0.00001), hematocrit percentage (43.06% vs. 40.14%; t = 5.9125, *p* < 0.00001), mean corpuscular volume (91.45 vs. 90.07; t = 2.2665, *p* = 0.02414), mean corpuscular hemoglobin (30.81 vs. 29.82; t = 3.9513, *p* = 0.00010), mean corpuscular hemoglobin concentration (33.69 vs. 33.16; t = 4.6862, *p* < 0.00001), plateletcrit (0.54 vs. 0.36; Z = 3.7999, *p* = 0.00014), and serum creatinine concentration (90.02 μmol/L vs. 73.60 μmol/L; Z = 6.5621, *p* < 0.00001). In contrast, men had significantly lower platelet count (239.97 vs. 267.73; t = –3.2261, *p* = 0.00139) and serum 25-hydroxyvitamin D3 concentration (28.96 ng/mL vs. 34.96 ng/mL; Z = –3.2759, *p* = 0.00105) compared with women.

**Table 5:** Comparison of biochemical parameters and SF-36 quality of life questionnaire scores between male and female orthopedic surgery patients.

Sex	Men = 113	Women = 187	Student’s *t*-Test/Mann–Whitney	*p*-Value
	M (SD)/[Q1:Q3] *	M (SD)/[Q1:Q3] *	U Test *	
Age	66.12 (9.80)	67.05 (10.26)	−0.7732	0.44000
BMI	29.94 (4.92)	28.71 (4.95)	2.0890	0.03755 #
WBC	7.22 (1.89)	6.86 (1.99)	1.5475	0.12280
LYM	1.73 (0.55)	1.70 (0.57)	0.4509	0.65236
NEU	4.72 (1.63)	4.50 (1.73)	1.1197	0.26376
MONO	0.57 (0.17)	0.49 (0.16)	3.8819	0.00013 #
EOS	0.13 [0.06:0.18]	0.12 [0.04:0.16]	1.4669 *	0.14238
BASO	0.04 [0.02:0.05]	0.04 [0.03:0.05]	0.8131 *	0.41613
HGB	14.51 (1.54)	13.40 (1.21)	6.9779	0.00001 #
HCT %	43.06 (4.22)	40.14 (4.10)	5.9125	0.00001 #
MCV	91.45 (5.23)	90.07 (5.07)	2.2665	0.02414 #
MCH	30.81 (2.00)	29.82 (2.17)	3.9513	0.00010 #
MCHC	33.69 (0.93)	33.16 (0.96)	4.6862	0.00000 #
PLT	239.97 (60.83)	267.73 (78.26)	−3.2261	0.00139 #
PDW	14.76 [10.70:13.40]	12.50 [11.10:13.50]	−0.7325 *	0.46384
MPV	10.35 [9.70:11.10]	12.24 [9.80:11.10]	−1.0091 *	0.31291
PCT	0.54 [0.21:0.28]	0.36 [0.23:0.33]	3.7999 *	0.00014 #
CRP	7.50 [0.85:4.09]	7.85 [1.07:4.83]	−1.0276 *	0.30411
25 (OH) D3	28.96 [19.10:34.40]	34.96 [22.40:42.90]	−3.2759 *	0.00105 #
HbA1c	5.82 [5.36:6.03]	5.92 [5.38:5.94]	0.4746 *	0.63505
Creatinine	90.02 [72.00:99.50]	73.60 [61.00:82.00]	6.5621 *	0.00001 #
SF-36	97.35 (10.52)	95.26 (9.93)	1.7282	0.08500

M—arithmetic mean; SD—standard deviation; Q1—lower quartile; Q3—upper quartile; *—variables do not follow a normal distribution according to the Kolmogorov–Smirnov test; Student’s *t*-test—applied for variables with a normal distribution; Mann–Whitney U test—applied for variables without a normal distribution—marked *; #—differences which are statistically significant (*p* < 0.05). **Abbreviations**: WBC, white blood cells; LYM, lymphocytes; NEU, neutrophils; MONO, monocytes; EOS, eosinophils; BASO, basophils; HGB, hemoglobin; HCT, hematocrit; MCV, mean corpuscular volume; MCH, mean corpuscular hemoglobin; MCHC, mean corpuscular hemoglobin concentration; PLT, platelets; PDW, platelet distribution width; MPV, mean platelet volume; PCT, plateletcrit; CRP, C-reactive protein; 25(OH)D_3_, 25-hydroxyvitamin D_3_; HbA1c, glycated hemoglobin; SF-36, 36-Item Short-Form Health Survey; BMI, body mass index.

Table 6 presents the results of multiple regression analysis, in which the rs2228570 *FokI* gene polymorphism, age, and body mass index were included as independent variables influencing biochemical parameters and SF-36 quality of life questionnaire scores among orthopedic surgery patients. A statistically significant model fit was obtained for the following parameters: white blood cell count, lymphocyte count, monocyte count, basophil count, hemoglobin concentration, hematocrit percentage, mean corpuscular volume, platelet count, plateletcrit, C-reactive protein, serum 25-hydroxyvitamin D3 concentration (ng/mL), serum creatinine concentration (μmol/L), and SF-36 score.

A higher white blood cell count was associated with the rs2228570 *FokI* CC genotype (ß = 0.52; *p* = 0.04350). A higher lymphocyte count was associated with the rs2228570 *FokI* CC genotype (ß = 0.26; *p* = 0.00025) and higher body mass index (ß = 0.01; *p* = 0.03855), whereas a lower lymphocyte count was associated with older age (ß = –0.01; *p* = 0.00089). A higher monocyte count was associated with higher body mass index (ß = 0.006; *p* = 0.00077). A higher hemoglobin concentration was associated with the rs2228570 *FokI* CC genotype (ß = 0.57; *p* = 0.00197), whereas a lower hemoglobin concentration was associated with older age (ß = –0.01; *p* = 0.00089). A higher hematocrit percentage was associated with the rs2228570 *FokI* CC genotype (ß = 1.42; *p* = 0.01102), the *FokI* TT genotype (ß = 1.45; *p* = 0.03539), and higher body mass index (ß = 0.15; *p* = 0.00340). A higher C-reactive protein level was associated with the rs2228570 *FokI* TT genotype (ß = 10.90; *p* = 0.00329). A higher serum creatinine concentration (μmol/L) was associated with older age (ß = 0.58; *p* = 0.00010) and higher body mass index (ß = 0.72; *p* = 0.01555). Additionally, a borderline significant association was observed between the rs2228570 *FokI* TT genotype and lower creatinine concentration (ß = –6.99; *p* = 0.08999). A higher SF-36 quality of life score was associated with the rs2228570 *FokI* TT genotype (ß = 6.31; *p* = 0.00009) and younger age (ß = –0.15; *p* = 0.00825).

A lower basophil count was associated with older age (ß = –0.0002; *p* = 0.02634). A lower mean corpuscular volume was associated with the rs2228570 *FokI* TT genotype (ß = –1.63; *p* = 0.04909). A lower platelet count was associated with older age (ß = –0.94; *p* = 0.02586). A lower plateletcrit was also associated with older age (ß = –0.02; *p* = 0.02781). A lower serum 25-hydroxyvitamin D3 concentration (ng/mL) was associated with higher body mass index (ß = –0.49; *p* = 0.01186), with a borderline significant association observed for the rs2228570 *FokI* CC genotype (ß = –4.12; *p* = 0.05653) and with younger age (ß = 0.35; *p* = 0.00026).

**Table 6:** Multiple regression model for biochemical parameters and SF-36 quality of life questionnaire scores (dependent variables). Independent variables include the rs2228570 *FokI* polymorphism (dummy variables, with the C/T genotype as the reference category), age, and body mass index.

β (*p*)	Reference β (−95%CI:+95%CI) *p* Value	rs2228570 C/C β (−95%CI:+95%CI) *p* Value	rs2228570 T/T β (−95%CI:+95%CI) *p* Value	Age β (−95%CI:+95%CI) *p* Value	BMI β (−95%CI:+95%CI) *p* Value
WBC	6.34 (4.36:8.33) *p* < 0.00001 #	0.52 (0.02:1.02) *p* = 0.04350 #	0.17 (−0.45:0.79) *p* = 0.58588	−0.01 (−0.03:0.01) *p* = 0.38036	0.04 (−0.01:0.08) *p* = 0.095135
LYM	1.94 (1.39:2.49) *p* < 0.00001 #	0.26 (0.12:0.40) *p* = 0.00025 #	−0.01 (−0.18:0.16) *p* = 0.88507	−0.01 (−0.02:−0.004) *p* = 0.00089 #	0.01 (0.001:0.03) *p* = 0.03855 #
NEU	3.93 (2.20: 5.65) *p* = 0.000011 #	0.25 (−0.19: 0.68) *p* = 0.262402	0.23 (−0.30:0.77) *p* = 0.39392	0.001 (−0.02: 0.02) *p* = 0.909983	0.02 (−0.02: 0.05) *p* = 0.425531
MONO	0.29 (0.12:0.46) *p* = 0.00096 #	0.005 (−0.04:0.05) *p* = 0.80664	−0.02 (−0.07:0.03) *p* = 0.49072	0.0005 (−0.001: 0.002) *p* = 0.57427	0.006 (0.003:0.01) *p* = 0.00077 #
EOS	0.03 (−0.07: 0.13) *p* = 0.57835	−0.005 (−0.03: 0.02) *p* = 0.73000	−0.03 (−0.06: 0.004) *p* = 0.09000	0.0001 (−0.001: 0.001) *p* = 0.80751	0.003 (0.001: 0.005) *p* = 0.01002 #
BASO	0.04 (0.02: 0.06) *p* = 0.00001 #	−0.001 (−0.005: 0.003) *p* = 0.63340	0.004 (−0.001: 0.01) *p* = 0.10351	−0.0002 (−0.0004: −0.00003) *p* = 0.02634 #	0.0003 (−0.00005: 0.0007) *p* = 0.08421
HGB	14.02 (12.58: 15.45) *p* < 0.00001 #	0.57 (0.21: 0.94) *p* = 0.00197 #	0.39 (−0.05: 0.84) *p* = 0.08465	−0.02 (−0.03: −0.004) *p* = 0.01695 #	0.03 (−0.003: 0.06) *p* = 0.07944
HCT %	38.94 (34.60: 43.27) *p* = <0.00001 #	1.42 (0.33: 2.52) *p* = 0.01102 #	1.45 (0.10: 2.80) *p* = 0.03539 #	−0.04 (−0.09: 0.008) *p* = 0.10064	0.15 (0.05: 0.24) *p* = 0.00340 #
MCV	88.48 (83.26: 93.69) *p* < 0.00001 #	−0.33 (−1.65: 0.99) *p* = 0.62503	−1.63 (−3.26: −0.007) *p* = 0.04909 #	0.05 (−0.005: 0.11) *p* = 0.07325	−0.04 (−0.15: 0.08) *p* = 0.55372
MCH	30.22 (28.03: 32.41) *p* < 0.00001 #	0.05 (−0.51: 0.60) *p* = 0.86532	−0.58 (−1.26: 0.10) *p* = 0.09485	0.01 (−0.01: 0.04) *p* = 0.38150	−0.02 (−0.07: 0.03) *p* = 0.36175
MCHC	34.19 (33.19: 35.19) *p* < 0.00001 #	0.12 (−0.13: 0.37) *p* = 0.34819	−0.11 (−0.42: 0.20) *p* = 0.49025	−0.01 (−0.01: 0.003) *p* = 0.14624	−0.01 (−0.03: 0.01) *p* = 0.35963
PLT	327.26 (253.06: 401.45) *p* < 0.00001 #	−6.51 (−25.25: 12.23) *p* = 0.49477	9.04 (−14.07: 32.16) *p* = 0.44175	−0.94 (−1.76: −0.11) *p* = 0.02586 #	−0.23 (−1.91: 1.44) *p* = 0.78463
PDW	11.43 (−6.34: 29.21) *p* = 0.20656	0.48 (−4.01: 4.97) *p* = 0.83289	5.50 (−0.04: 11.04) *p* = 0.05146	−0.09 (−0.28: 0.11) *p* = 0.37067	0.23 (−0.17: 0.63) *p* = 0.25284
MPV	15.50 (−3.50: 34.50) *p* = 0.10935	3.54 (−1.25: 8.34) *p* = 0.147205	−0.24 (−6.16: 5.67) *p* = 0.93489	−0.05 (−0.26: 0.16) *p* = 0.61753	−0.05 (−0.48: 0.38) *p* = 0.81388
PCT	2.14 (0.52: 3.75) *p* = 0.00994 #	0.002 (−0.41: 0.41) *p* = 0.99277	0.03 (−0.47: 0.54) *p* = 0.89422	−0.02 (−0.04: −0.002) *p* = 0.02781 #	−0.01 (−0.05: 0.02) *p* = 0.49914
CRP	8.00 (−15.24: 31.25) *p* = 0.49850	0.40 (−5.47: 6.27) *p* = 0.89372	10.90 (3.66: 18.14) *p* = 0.00329 #	0.06 (−0.19: 0.32) *p* = 0.63226	−0.22 (−0.75: 0.30) *p* = 0.40383
25 (OH) D_3_	24.57 (7.79: 41.35) *p* = 0.00424 #	−4.12 (−8.36: 0.12) *p* = 0.05653 ◊	1.71 (−3.51: 6.94) *p* = 0.51868	0.35 (0.16: 0.54) *p* = 0.00026 #	−0.49 (−0.87: −0.11) *p* = 0.01186 #
HbA1c	4.10 (2.58: 5.62) *p* < 0.00001	−0.05 (−0.43: 0.34) *p* = 0.81522	−0.26 (−0.73: 0.21) *p* = 0.28222	0.01 (−0.004: 0.02) *p* = 0.13562	0.03 (−0.0008: 0.07) *p* = 0.05558 ◊
Creatinine	20.97 (−4.99: 46.93) *p* = 0.11298	0.56 (−5.99: 7.12) *p* = 0.86685	−6.99 (15.08: 1.10) *p* = 0.08999 ◊	0.58 (0.29: 0.87) *p* = 0.00010 #	0.72 (0.14: 1.31) *p* = 0.01555 #
SF-36	99.46 (89.44: 109.49) *p* < 0.00001 #	1.35 (−1.18: 3.89) *p* = 0.29368	6.31 (3.19: 9.43) *p* = 0.00009 #	−0.15 (−0.26: −0.04) *p* = 0.00825 #	0.17 (−0.05: 0.40) *p* = 0.12915

Reference—indicating the reference category in the regression model; β—regression coefficient; CI—confidence interval (−95% CI; +95% CI); *p*—level of statistical significance; #—differences which are statistically significant (*p* < 0.05); ◊—differences bordering on statistical significance. **Abbreviations**: WBC, white blood cells; LYM, lymphocytes; NEU, neutrophils; MONO, monocytes; EOS, eosinophils; BASO, basophils; HGB, hemoglobin; HCT, hematocrit; MCV, mean corpuscular volume; MCH, mean corpuscular hemoglobin; MCHC, mean corpuscular hemoglobin concentration; PLT, platelets; PDW, platelet distribution width; MPV, mean platelet volume; PCT, plateletcrit; CRP, C-reactive protein; 25(OH)D_3_, 25-hydroxyvitamin D_3_; HbA1c, glycated hemoglobin; SF-36, 36-Item Short-Form Health Survey; BMI, body mass index.

## 4. Discussion

The present study shows that, while rs2228570 (*FokI*) genotype distributions did not differ between orthopedic patients and controls and conformed to Hardy–Weinberg expectations, genotype status in patients was associated with several hematologic and inflammatory readouts after adjustment for age and BMI. Specifically, the CC genotype related to higher white blood cell and lymphocyte counts (β = 0.52 × 10^9^/L, *p* = 0.044; β = 0.26 × 10^9^/L, *p* < 0.001), as well as higher hemoglobin and hematocrit (β = 0.57 g/dL, *p* = 0.002; β = 1.42 pp, *p* = 0.011). In turn, TT was associated with lower MCV (β = −1.63 fL, *p* = 0.049), higher C-reactive protein (CRP; β = 10.90 mg/L, *p* = 0.003), and—together with CC—higher hematocrit (TT: β = 1.45 pp, *p* = 0.035; CC: β = 1.42 pp, *p* = 0.011), as well as higher SF-36 scores (β = 6.31, *p* < 0.001). By contrast, circulating 25-hydroxyvitamin D_3_ did not differ significantly by genotype (borderline lower values for CC; β = −4.12 ng/mL, *p* = 0.057), and in this cohort was positively associated with age and negatively associated with BMI, patterns that likely reflect cohort-specific environmental and metabolic influences [20]. These patterns were observed on the background of stable genotype frequencies across clinical strata such as sex, smoking, hypertension, and diabetes.

Mechanistically, rs2228570 shifts the translation start site of *VDR*, yielding a shorter 424-aa isoform for the C allele and a longer 427-aa isoform for the T allele [10,17]. In this context, the TT–CRP linkage may reflect relatively weaker *VDR*-mediated immunomodulation and heightened acute-phase signaling [1,2], whereas the CC profile—higher WBC/LYM and erythroid indices—may result from a more active *VDR* signaling milieu [10,17]. The lack of a clear genotype effect on serum 25(OH)D_3_ aligns with the notion that circulating vitamin D levels are strongly shaped by external exposures (sunlight, supplementation) and adiposity [1,2], and that rs2228570 may act primarily at the tissue-response level rather than by determining ligand availability.

The analysis of health-related quality of life (SF-36) reveals an intriguing paradox. The statistically significant association of the TT genotype with higher SF-36 scores, despite being linked to a higher level of an inflammatory marker (CRP), suggests that our current understanding of the *FokI* polymorphism’s phenotypic effects is incomplete [21]. This divergence between objective biomarkers and subjective health perception may reflect domain-specific effects of the TT genotype on psychological well-being or resilience [13,17,22,23]. Future studies should conduct a domain-level analysis of the SF-36, rather than just an overall score, to identify which specific aspects of quality of life are modulated by this polymorphism [24]. The combined assessment of *VDR* genotype, objective biomarkers (such as CRP), and subjective quality-of-life indicators (SF-36) could provide a more personalized and holistic patient evaluation [11,17,19,25], enabling better tailored perioperative care and rehabilitation.

Clinically, the magnitude of the TT–CRP difference (≈11 mg/L) is potentially meaningful for perioperative risk profiling, and the fact that signals are detectable in first-line tests (complete blood count, CRP) favors translational applicability. Key covariates (age, BMI) demonstrated systematic associations with several endpoints, underscoring the importance of covariate control and the possibility of residual confounding despite adjustment [26]. Future studies should incorporate homogeneous surgical indications, seasonality and supplementation data for vitamin D, and extended biomarker panels (e.g., 1,25(OH)_2_D_3_, vitamin D binding protein, cytokines) to clarify mechanism and clinical utility. Nonetheless, our findings indicate that *VDR* genotyping may complement routine laboratory measures for personalized perioperative assessment [11,17,27]. Although our study did not directly assess musculoskeletal recovery outcomes, the observed associations with hematologic and inflammatory markers suggest potential downstream implications for postoperative rehabilitation. Beyond the primary associations, our data situate rs2228570 within a broader framework in which vitamin D signaling shapes immune–inflammatory tone and hematopoiesis at the tissue level rather than dictating circulating 25(OH)D_3_ [28,29,30].

This study has several limitations. Heterogeneity in surgical indications, preoperative status, and other unmeasured factors may have influenced inflammatory readouts, and residual confounding cannot be excluded despite adjustment for age, BMI, and comorbidities. Additional determinants of vitamin D biology—such as seasonality, supplementation, and vitamin D-binding protein may have influenced genotype–ligand associations [13,25,31,32,33,34]. Future studies should adopt prospective designs with homogeneous surgical indications, record seasonality and supplement use, and expand biomarker panels (e.g., 1,25(OH)_2_D_3_, vitamin D-binding protein, and cytokines such as IL-6 and TNF-α) to clarify mechanisms and clinical utility. Incorporating *VDR* genotyping into interventional studies may explain interindividual variability in response to vitamin D and help refine personalized nutritional and rehabilitation protocols.

By integrating genetic, biochemical, and patient-reported outcomes, our study underscores both the potential and the challenges of translating vitamin D biology into personalized perioperative care, in line with the broader controversies highlighted in the current debate on vitamin D. Clinically, our findings suggest that VDR rs2228570 genotyping could complement routine laboratory tests such as CRP and complete blood count in perioperative care [35]. Identifying patients with a heightened inflammatory tone or distinct hematologic profiles may help tailor monitoring intensity, optimize perioperative management, and personalize rehabilitation strategies. This translational potential highlights the added value of genetic markers in everyday orthopedic practice for risk stratification and personalized rehabilitation.

## 5. Conclusions

Our study shows that the *VDR* rs2228570 polymorphism is associated with distinct perioperative inflammatory and hematological profiles, suggesting that this genetic variant may influence patient responses in orthopedic settings. Specifically, carriers of the TT genotype exhibited higher CRP levels but also reported better self-assessed quality of life, underscoring the complex interplay between biological markers and psychosocial outcomes. In addition, both CC and TT homozygotes were associated with higher hematocrit, pointing to genotype-specific effects on erythropoiesis. These findings highlight the potential of integrating genetic information, such as *VDR* genotyping, with routine laboratory tests (e.g., CRP, CBC) to refine perioperative risk stratification and postoperative care. Importantly, they also support the view that receptor-level variation contributes to heterogeneity in clinical responses to vitamin D and should be considered in supplementation and rehabilitation strategies. Future studies with larger, multi-center cohorts and functional assays are warranted to validate these associations, clarify underlying mechanisms, and explore their implications for personalized nutritional and clinical strategies, including genotype-informed approaches to vitamin D supplementation and rehabilitation in surgical patients.

## Figures and Tables

**Table 1:** Hardy–Weinberg equilibrium analysis for orthopedic surgery patients and the control group, calculated with bias-adjusted HWE software.

rs2228570 *FokI*		Observed (Expected)	Allele Freq	χ^2^ *p* Value
Patients undergoing orthopedic surgery; n = 300	CC	97 (99.2)	p (C) = 0.57 q (T) = 0.43	0.2671; *p* = 0.6053
	CT	151 (146.6)		
	TT	52 (54.2)		
Control; n = 200	CC	61 (62.2)	p (C) = 0.56 q (T) = 0.44	0.1108; *p* = 0.7692
	CT	101 (98.7)		
	TT	38 (39.2)		

*p*–statistical significance χ^2^ test.

**Table 2:** Comparison of the rs2228570 *FokI* polymorphism between orthopedic surgery patients and the control group.

	Patients Undergoing Orthopedic Surgery; n = 300	Control; n = 200	χ^2^; *p* Value
CC—n (%)	97 (32.33%)	61 (30.50%)	0.3134; *p* = 0.8549
CT—n (%)	151 (50.33%)	101 (50.50%)	
TT—n (%)	52 (17.33%)	38 (19.00%)	
C—n (%)	345 (57.5%)	223 (55.75%)	0.2995; *p* = 0.5842
T—n (%)	255 (42.5%)	177 (44.25%)	

*p*–statistical significance χ^2^ test.

**Table 3:** Comparison of the rs2228570 *FokI* polymorphism in orthopedic surgery patients according to sex, smoking status, hypertension, and diabetes mellitus.

Sex	M = 113	K = 187	χ^2^; *p* Value
CC N (%)	40 (35.40%)	57 (30.48%)	0.9645; *p* = 0.61744
CT N (%)	53 (46.90%)	98 (52.41%)	
TT N (%)	20 (17.70%)	32 (17.11%)	
**Smoking status**	**Yes = 31**	**No = 269**	1.6151; *p* = 0.4460
CC N (%)	12 (38.71%)	85 (31.60%)	
CT N (%)	16 (51.61%)	135 (50.19%)	
TT N (%)	3 (9.68%)	49 (18.22%)	
**Hypertension**	**Yes = 197**	**No = 103**	2.7394: *p* = 0.2542
CC N (%)	66 (33.50%)	31 (30.10%)	
CT N (%)	102 (51.78%)	49 (47.57%)	
TT N (%)	29 (14.72%)	23 (22.33%)	
**Diabetes mellitus**	**Yes = 59**	**No = 241**	0.2426; *p* = 0.8857
CC N (%)	20 (33.90%)	77 (31.95%)	
CT N (%)	30 (50.85%)	121 (50.21%)	
TT N (%)	9 (15.25%)	43 (17.84%)	

## Data Availability

The data presented in this study are available on request from the corresponding author. The data are not publicly available due to privacy concerns.
